# Path planning for endovascular catheterization under curvature constraints via two-phase searching approach

**DOI:** 10.1007/s11548-021-02328-x

**Published:** 2021-03-11

**Authors:** Zhen Li, Jenny Dankelman, Elena De Momi

**Affiliations:** 1 NearLab, Department of Electronics, Information and Bioengineering Department (DEIB), Politecnico di Milano, Milan, Italy; 2 Department of Biomechanical Engineering, Delft University of Technology, Delft, Netherlands

**Keywords:** Path planning, Flexible catheter, Autonomous endovascular intervention, Curvature constraints, Robotic surgery

## Abstract

**** Purpose**:**

Planning a safe path for flexible catheters is one of the major challenges of endovascular catheterization. State-of-the-art methods rarely consider the catheter curvature constraint and reduced computational time of path planning which guarantees the possibility to re-plan the path during the actual operation.

**** Methods**:**

In this manuscript, we propose a fast two-phase path planning approach under the robot curvature constraint. Firstly, the vascular structure is extracted and represented by vascular centerlines and corresponding vascular radii. Then, the path is searched along the vascular centerline using breadth first search (BFS) strategy and locally optimized via the genetic algorithm (GA) to satisfy the robot curvature constraint. This approach (BFS-GA) is able to respect the robot curvature constraint while keeping it close to the centerlines as much as possible. We can also reduce the optimization search space and perform parallel optimization to shorten the computational time.

**** Results**:**

We demonstrate the method’s high efficiency in two-dimensional and three-dimensional space scenarios. The results showed the planner’s ability to satisfy the robot curvature constraint while keeping low computational time cost compared with sampling-based methods. Path replanning in femoral arteries can reach an updating frequency at $6.4\pm 2.3$Hz.

**** Conclusion**:**

The presented work is suited for surgical procedures demanding satisfying curvature constraints while optimizing specified criteria. It is also applicable for curvature constrained robots in narrow passages.

## Introduction

**Fig. 1:**
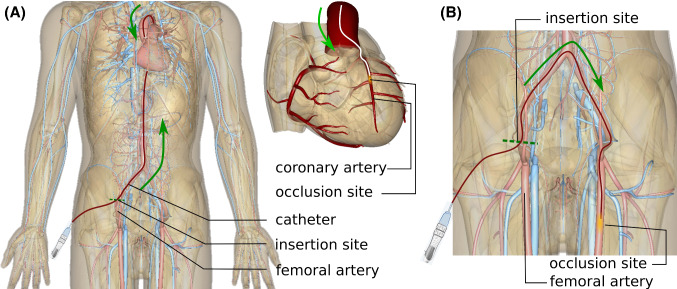
Clinical background (A) coronary endovascular procedure, (B) femoral endovascular procedure (The anatomy models are made using BodyParts3D, ©2008 The Database Center for Life Science licensed under CC Attribution-Share Alike 2.1 Japan)

Percutaneous coronary intervention (PCI) is used to widen stenotic and occluded blood vessels by pushing the plaque aside and placing a stent nearby to restore and maintain the blood circulation. For example in Fig. 1, a catheter is inserted from a femoral artery and targeting the occlusion site.

Tool and navigation guidance can lower the skill requirements for percutaneous treatment. Nowadays, steerable catheters have been developed via mechanical, magnetic, and fluidic actuation principles. Steerable catheters have different bending capabilities exhibiting a minimum bending radius. The minimum bending radius found in literature lies between 8.13mm and 171mm [17].

Path planning is one of the major challenges of endovascular catheterization. Vascular centerlines were seen as a reference trajectory, and centerline extraction has aroused the interest of researchers. A graph matching method is proposed to establish the correspondence between the 3D pre-operative and 2D intra-operative skeletons extracting from fluoroscopic images, and then, the two skeletons are registered by skeleton deformation [19]. Nevertheless, the path planning approach which merely follows centerlines might be infeasible when the path curvature exceeds catheters bending capability. For example, if the robot is attempting to follow the centerlines (like in [19]), the minimum bending radius is less than 1mm at the bifurcation (Fig. 1B), which exceeds the robot bending capability 13.1mm [1] and makes the robot fail to follow.

A performant path planner should provide a reliable path within the catheter capability. Sampling-based methods such as extended probabilistic roadmap [8] and bidirectional rapidly exploring random tree (Bi-RRT) [5] are able to plan the path in configuration space. These methods have been coupled with Dubins path and Bézier spline to generate curvature bounded paths. Adaptive fractal tree (AFT) [13] takes advantage of the fractal theory and the architecture of graphics processing units (GPUs) paralleling the planning process. It has a higher success rate than RRTs, as demonstrated for needle insertions in a complex environment [13]. However, the success rate of RRTs or AFT is not always ensured.

To overcome the drawbacks mentioned above, a compromise between following the vascular centerlines and satisfying the curvature constraint is needed. An approach that simply decreases the path arch height at the $180^{\circ }$ turning until the curvature constraint is satisfied was implemented [18]. In [6], an ant colony optimization method was presented with an average time cost of 12.3s (min 2, max 30). Also in [14], a backbone curve method was implemented to optimize the path under kinematic analysis for a cable-driven continuum robot in a cardiovascular system. Nevertheless, this work considers the constrained optimization problem along the overall path without reducing the optimization search space.

More importantly, reducing computational time would help path planners to be applied in path replanning. Intra-operatively, planned paths might be infeasible or less accurate due to environment deformations and sensing uncertainties. The work in [7] quantified the displacement of arteries during endovascular catheterization: The aortic bifurcation was mostly displaced in a cranial direction with the median cranio-caudal dislocation of 6.7mm (min 2.1, max 12.3). Considering that the high computational time of 12.3s [6] can barely make the path adapted to the deformation, the need for real-time path planning with low computational time is highlighted. In real applications, the proper replanning frequency is usually constrained by multiple factors: the catheter tip position tracking frequency, vision sensing feedback frequency, and controller frequency. For example, the frequency of an electromagnetic tracking system (Aurora) is 40Hz [11], the frequency of intra-operative model reconstruction is 1.25Hz [19], and the controller frequency is 10Hz [15]. Therefore, the replanning frequency needs to be set accordingly.

In this manuscript, we propose a fast two-phase path planning approach considering the robot curvature and time constraints.

## Methodology

**Fig. 2:**
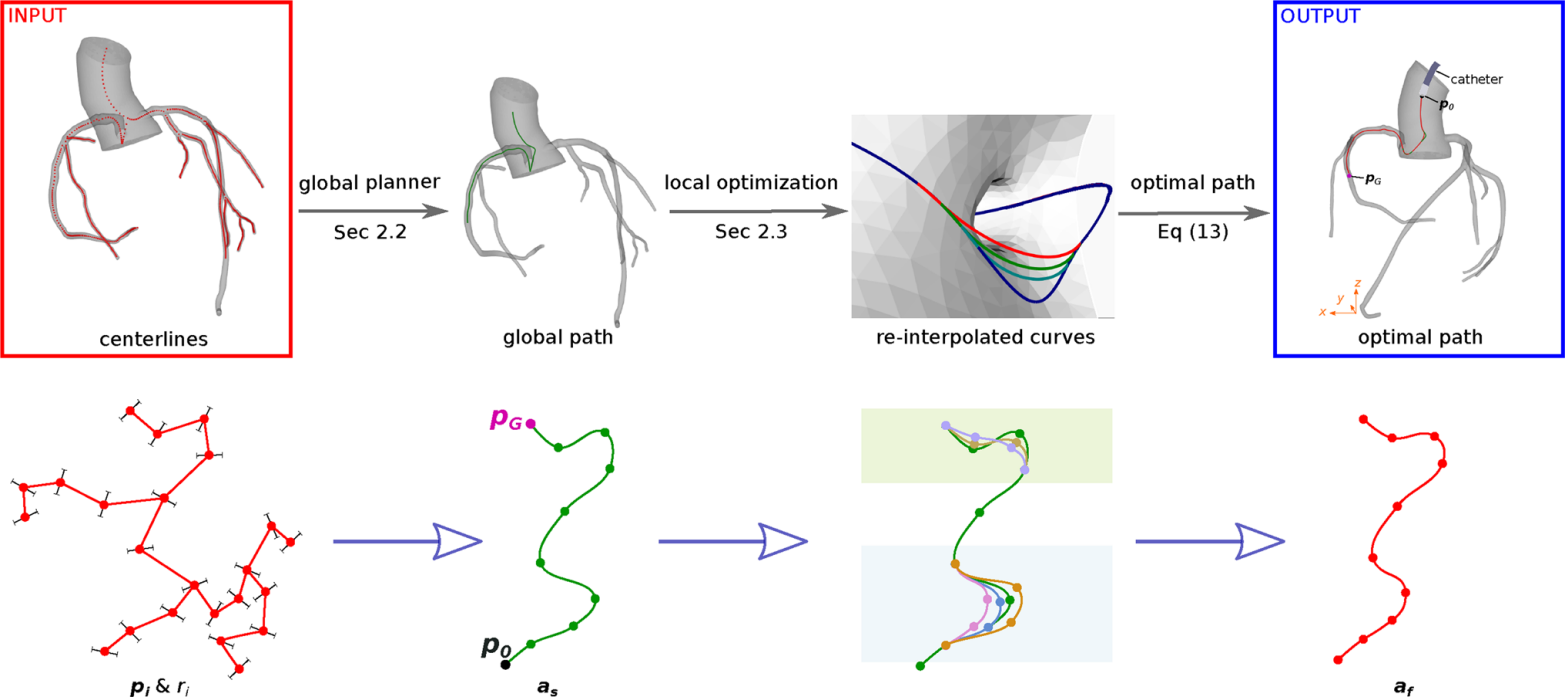
Pipeline for the proposed path planning approach: given centerlines and radii, global planner computes a tentative curve, then local planner optimizes the curve to satisfy the catheter curvature constraint

The proposed approach is a two-phase searching framework (see pipeline in Fig. 2). The inputs of the path planner are the centerline points ${\varvec{p_i}}$ and their minimum distances to the vascular walls $r_i$, where *i* is the running index (detailed in Sec 2.1). Globally, we find a cubic B-spline curve ${\varvec{a_s}}$ along the vascular centerlines from a user-defined initial point ${\varvec{p_0}}$ to a goal point ${\varvec{p_G}}$ (detailed in Sec 2.2). Locally, the aforementioned curve is optimized to satisfy the catheter curvature constraint. The final output curve ${\varvec{a_f}}$ is the curve with locally optimized curve segments (detailed in Sec 2.3).

### Centerline extraction

**Fig. 3:**
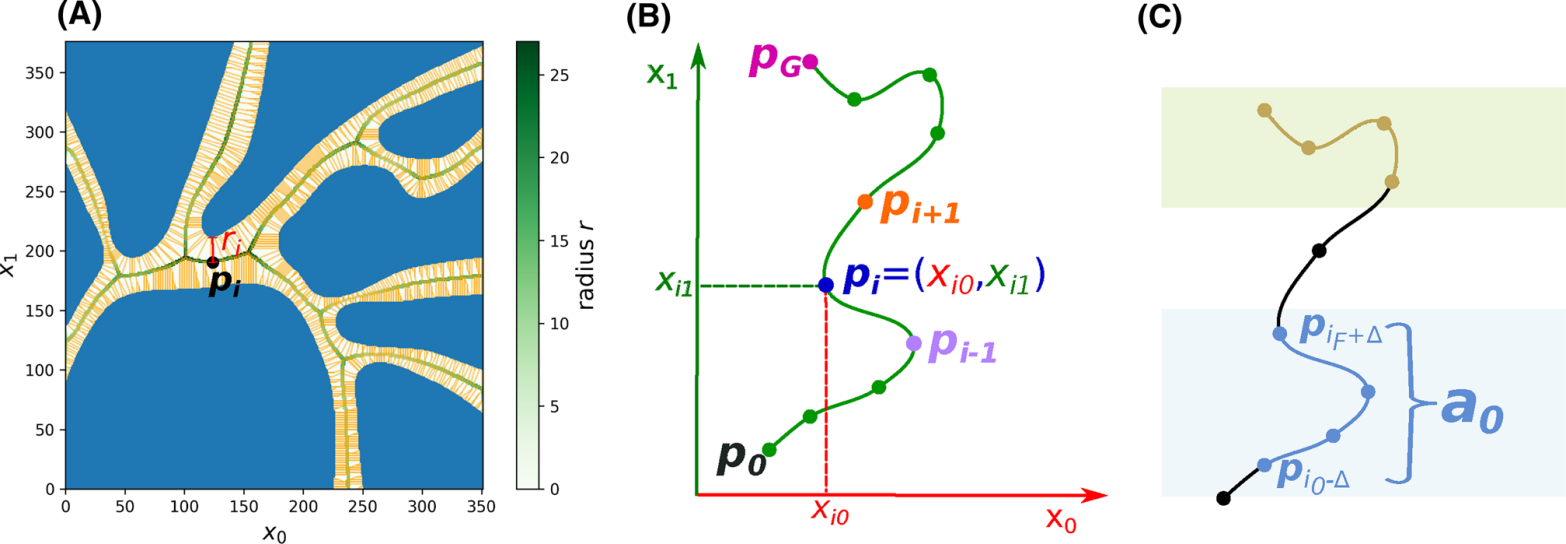
The schematic view of: (A) Voronoi regions to extract centerlines and radii, where Voronoi vertices, Voronoi edges, and centerlines are blue, yellow, and green, respectively. (B) Example of path points definition (C) Example of curve segments to be optimized

Our approach employs the method demonstrated in [2], which treats the centerlines as the minimal action paths linking Voronoi vertices inside the model surface. By solving a nonlinear hyperbolic equation (Eikonal equation) followed by an ordinary differential equation, the approach [2] provides the minimal action paths points ${\varvec{p_i}}$ that locally maximize their minimum distances $r_i$ to the boundary of the surface. The vascular modeling toolkit (VMTK) library based on [2] was used to automatically extract ${\varvec{p_i}}$ and $r_i$. For example, Fig 3A shows the Voronoi regions with Voronoi vertices (blue), Voronoi edges (yellow), and extracted centerline points (green).

### Global planner

From the global planner, a tentative curve from an initial point ${\varvec{p_0}}$ to a goal point ${\varvec{p_G}}$ inside blood vessels is obtained (see Fig 3B). Each waypoint is presented in the N-dimensional Cartesian coordinate system ($N=2$ or 3).

1 $$\begin{aligned} {\varvec{p_i}}=[x_{i0}, x_{i1}, \cdots , x_{i(N-1)}] \quad i=0,\cdots ,G \end{aligned}$$

The initial point ${\varvec{p_0}}$ is regarded as the exploration tree root. The breadth first search (BFS) strategy starts at the tree root and explores the k-nearest neighbor centerline nodes at the present depth prior to moving on to the nodes at the next depth level. It stops when the goal point is visited. Thus a list of centerline points from ${\varvec{p_0}}$ to ${\varvec{p_G}}$ is obtained by navigating through the BFS tree.

The list of points is smoothed via cubic B-spline interpolation and a tentative B-spline curve ${\varvec{a_s}}$ is then obtained. Given a knot sequence $t_0,\cdots ,t_{G}$, B-splines with degree $M=3$ can be defined by the Cox-de Boor recursion formula as (2), where *w* is the parametric space of the B-spline.

2 $$\begin{aligned} \begin{aligned} {}^0{\varvec{p_i}}(w) =&{\left\{ \begin{array}{ll}1 &{} t_i \le w < t_{i+1} \\ 0 &{} \text {otherwise} \end{array}\right. }\\ {}^{M}{\varvec{p_i}}(w) =&\frac{w-t_i}{t_{i+M}-t_i} {}^{M-1}{\varvec{p_i}}(w) + \frac{t_{i+M+1}-w}{t_{i+M+1}-t_{i+1}} {}^{M-1}{\varvec{p_{i+1}}}(w) \end{aligned} \, i=0,\cdots ,G \end{aligned}$$

### Local planner

The curvature $s_i$ at ${\varvec{p_i}}$ along the B-spline interpolated curve is defined as (3) in a generic form. Specifying $N=3$, the expression is simplified as (4).

3 $$\begin{aligned} s_i&= \frac{\sqrt{\frac{1}{2} \sum \limits _{\scriptscriptstyle j=0}^{\scriptscriptstyle N-1} \sum \limits _{\scriptscriptstyle k=0}^{\scriptscriptstyle N-1} ({\dot{x}}_{ij}{\ddot{x}}_{ik}-{\dot{x}}_{ik}{\ddot{x}}_{ij})^2}}{(\sum \limits _{\scriptscriptstyle j=0}^{\scriptscriptstyle N-1} {\dot{x}}_{ij}^2 )^{\frac{3}{2}}} \quad i=0,\cdots ,G \end{aligned}$$

4 $$\begin{aligned} s_i&= \frac{\sqrt{({\dot{x}}_{i0}{\ddot{x}}_{i1}-{\dot{x}}_{i1}{\ddot{x}}_{i0})^2+({\dot{x}}_{i0}{\ddot{x}}_{i2}-{\dot{x}}_{i2}{\ddot{x}}_{i0})^2+({\dot{x}}_{i1}{\ddot{x}}_{i2}-{\dot{x}}_{i2}{\ddot{x}}_{i1})^2}}{({\dot{x}}_{i0}^2+{\dot{x}}_{i1}^2+{\dot{x}}_{i2}^2)^{\frac{3}{2}}} \end{aligned}$$

The curvature constraint is expressed as (5), and *S* is the allowed maximal curvature value depending on robot kinematic constraints.

5 $$\begin{aligned} s_i \le S \text { for } i=0,\cdots ,G \end{aligned}$$

It is evaluated for the tentative curve ${\varvec{a_s}}$ firstly. If the constraint is satisfied, ${\varvec{a_s}}$ will be the final path without further optimization. Otherwise, local optimization will be applied. The curve to be optimized ${{\varvec{a}}}_0$ is defined as

6 $$\begin{aligned} \begin{aligned} \text {If } s_i>S \text { for } i=i_0,\cdots ,i_F \\ \text {Then } {{\varvec{a}}}_0=[{{\varvec{p}}}_{i_0-\varDelta }, \cdots , {{\varvec{p}}}_{i_F+\varDelta }] \end{aligned} \end{aligned}$$

where ${{\varvec{a}}}_0$ is the curve segment exceeding robot bending capability, and it is represented by a list of waypoints (see Fig. 3C). $\varDelta$ is the user-defined marginal capacity for local optimization, such as $5\%$ of the total number of path points. If the initial or goal point is included in ${{\varvec{a}}}_0$, its pose will also be optimized.

**Fig. 4:**
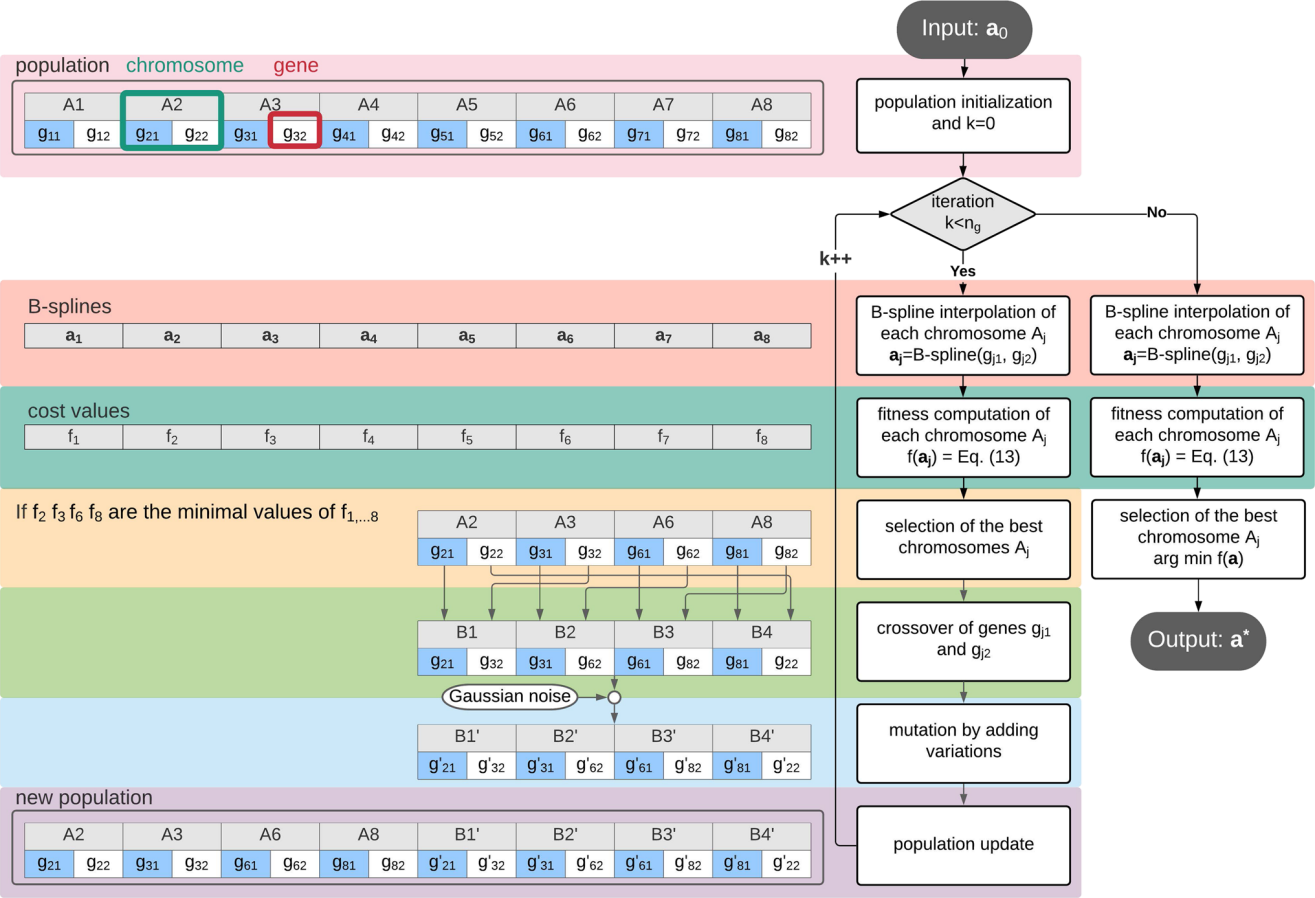
Schematics of the genetic algorithm procedure for local optimization

Genetic algorithm (GA) finds the optimal re-interpolated curve segment. In Fig. 4, there are 8 chromosomes making up the initial population. Each chromosome $A_j(j=1\cdots 8)$ is composed of 2 genes, which are the parameters determining the re-interpolated B-spline curve ${\varvec{a_j}}$. Specifically, the gene $g_{j1}$ is the number of points that are assigned with weight 0 when performing B-spline fitting; the gene $g_{j2}$ is the smoothness value that affects the trade-off between smoothness and displacement during spline fitting, and it is the upper boarder of the error sum of displacement squares. Then the fitness $f_j$ is computed for each re-interpolated curve segment according to a cost function. Next, the best re-interpolated curves are selected for mating. For example, there are 4 chromosomes selected for mating in Fig. 4. Then the crossover and mutation of genes are performed so that the population is updated. During mutation, a Gaussian distributed noise $\varDelta g_j \sim \mathcal {N}(\mu ,\,\sigma ^{2})$ is added to the genes. Finally, the optimal curve segment is selected from the population after $n_g$ iterations.

The cost function is designed to find the optimal path by a trade-off between the distance to vascular walls, path length, and curvature. The constrained optimization problem is formulated as

7 $$\begin{aligned} \begin{aligned} \min f({\varvec{a}})&= w_1 g_d({\varvec{a}}) + w_2 g_s({\varvec{a}})+ w_3 g_l({\varvec{a}}) \\ \text {s.t. }&s_i\le S \text { for } i=i_0-\varDelta ,\cdots ,i_F+\varDelta \\&d_i \le r_i \text { for } i=i_0-\varDelta ,\cdots ,i_F+\varDelta \end{aligned} \end{aligned}$$

where $g_d({\varvec{a}})$ is the mean value of normalized distances to the centerlines, $g_s({\varvec{a}})$ is the mean value of normalized curvatures, and $g_l({\varvec{a}})$ is the normalized path length.

The mean value of the normalized distances to the centerlines $g_d({\varvec{a}})$ is

8 $$\begin{aligned} g_d({\varvec{a}})&= \text {mean}\left( d_c/r\right) \end{aligned}$$

9 $$\begin{aligned} d_{ci}&=\min _{j=0,\cdots , G}||{\varvec{p_j^{0}}}-{\varvec{p_i}}|| \quad i=i_0-\varDelta ,\cdots ,i_F+\varDelta \end{aligned}$$

where $d_c$ is the distance from B-spline curve ${\varvec{a}}$ to the centerline. At the i-th index, $d_{ci}$ is computed by the minimum value of Euclidean distance from the new point ${\varvec{p_i}}$ to centerline points ${\varvec{p_j^{0}}}$.

The mean value of normalized curvatures $g_s({\varvec{a}})$ is formulated as

10 $$\begin{aligned} g_s({\varvec{a}}) = \text {mean}(s)/S \end{aligned}$$

The normalized path length $g_l({\varvec{a}})$ is presented as (11), where the length $l({\varvec{a}})$ is a cumulative sum of the distance between adjacent points.

11 $$\begin{aligned} g_l({\varvec{a}})&=l({\varvec{a}})/l({\varvec{a_0}}) \end{aligned}$$

12 $$\begin{aligned} l({\varvec{a}})&=\sum _{\scriptscriptstyle i_0-\varDelta }^{\scriptscriptstyle i_F+\varDelta } || {\varvec{p_{i+1}}} - {\varvec{p_i}} || \end{aligned}$$

Moreover, there are two constraints in (7): the curvature constraint and the collision avoidance constraint. In the collision avoidance constraint, by keeping the distance smaller than the vascular radius, the point is ensured to locate inside blood vessels. Since the path points are already refined in the B-spline interpolation in (2), the collision avoidance constraint is checked merely for the path points to reduce computational time cost.

The constrained optimization problem (7) is converted to an unconstrained one via moving constraints to the objective function as

13 $$\begin{aligned} \begin{aligned} \min f({\varvec{a}})&= w_1 g_d({\varvec{a}}) + w_2 g_s({\varvec{a}})+ w_3 g_l({\varvec{a}}) \\&\quad + w_4\max \{0, s-S\} + w_5 \max \{0, d_{c} - r\} \end{aligned} \end{aligned}$$

Here, in order to satisfy the hard constraints (curvature constraint and collision avoidance), the weights assigned to the cost function should have a significant difference between $w_4, w_5$ and others, for example $w_1=1, w_2=1, w_3=1, w_4=1000, w_5=1000$. When the hard constraints are satisfied, the last two elements are 0, otherwise, a large number will be added to the cost function value $f({\varvec{a}})$, indicating that the corresponding solution ${\varvec{a}}$ will not be selected since the procedure intends to find the minimum cost value. After $n_g$ iterations, if the optimal cost value is greater than a reasonable threshold (such as 1000), which means the constraints are not fully satisfied, there is no feasible solution until now. To look for new solutions within the time limit, the number of iterations $n_g$ will be increased. If the time limit is reached and there is still no feasible solution found, the path planner fails to find a path respecting all constraints.

There may be several portions of the tentative curve exceeding the allowed maximum curvature. In that case, each portion is assigned to an individual local planning thread. Multiple threads are carried on in parallel, instead of being conducted in serial to reduce computational time. After all the threads are done, the final path ${\varvec{a_f}}$ under curvature constraint is obtained.

### Evaluation metrics

Multiple criteria are chosen for performance evaluation. The time cost *t* is the time spent on path planning in a single trail from start to finish. The path length (12) is one of the essential components to evaluate the path optimality, and it is normalized by dividing it with the shortest distance from the initial point to the goal point. The curvature (3) is used to evaluate the bending extent of a curve.

The minimum distance to vascular walls at point ${\varvec{p_i}}$ can be obtained by the subtraction of two elements: the vascular radius $r_i$ and the distance to vascular centerline $d_{ci}$ given in (9). The distance to the vascular wall represents a safety margin ensuring collision avoidance between the catheter tip and vascular wall. To prevent physical harms as scratching to soft tissues if the catheter comes in contact with vascular walls, the distance to the vascular wall should not be less than the outer radius of the catheter.

The success rate is defined as the fraction or percentage of success among a number of attempts as $\delta = n_s/n$, where $n_s$ is the successful times to find a path and *n* is the number of attempts. For the proposed two-phase searching approach in this manuscript, a feasible path solution can be found as long as there is a feasible solution between initial and goal points.

### Experimental setup

**Table 1:** The datasets description and related parameters of experiments

*dataset*	*subjects*	*tortuosity*	*source*	*S* (mm$^{-1}$)	*trials*
*G1*	5	$2.365\pm 0.100$	-	0.08	250
*G2*	3	$1.067\pm 0.015$	[3]	0.10	150
*G3*	4	$1.075\pm 0.045$	[4, 12, 16]	0.08	200
*G4*	1	$1.501\pm 0.120$	[16]	0.20	100

This work targets endovascular procedures such as PCI, EVAR, TAVI, and iliac recanalization. The datasets include models such as coronary artery, aorta, femoral artery, peripheral arterial, etc., to evaluate and validate the approach. The datasets are classified into 4 groups, including 2D (*G*1, *G*2) and 3D (*G*3, *G*4) space scenarios.

The dataset *G*1 contains 2D images describing femoral arteries (pixel resolution of $220\times 294$ and spacing of 0.68mm). The dataset *G*2 includes 2D images describing lower limb arteries (pixel resolution of $2822\times 1539$ and spacing of 0.37mm). The dataset *G*3 includes several 3D mesh models: (i) A model which takes patient-specific computed tomography (CT) images as inputs, typically in a $512\times 512\times 737$ voxel dimension with a voxel spacing of $0.6445\times 0.6445\times 0.8$mm; (ii) A model which takes patient-specific magnetic resonance imaging (MRI) images as inputs, typically in a $512\times 64\times 512$ voxel dimension with a voxel spacing of $0.7813\times 2.0\times 0.7813$mm; (iii) An embeddable model of the lower limb made from anatomical parts, with the physical dimension of $852\times 116\times 169$mm; (iv) A mesh model of a single femoral artery with a physical dimension of $37\times 88\times 450$mm. The dataset *G*4 includes a 3D mesh model describing coronary arteries in a physical dimension of $102\times 89\times 101$mm. Table 1 provides other information of the datasets, among which the tortuosity is used to measure the arc-chord ratio of vascular structure.

The inputs of the path planner are obtained as follows. First, the centerline is extracted using the VMTK module on the platform 3DSlicer. Second, Gaussian distributed noise $\varDelta {{\varvec{p}}}_{0,G} \sim \mathcal {N}(\mu =0,\,\sigma =10)$ is added to the initial and goal points in each trail to increase data variability. Third, without loss of generality, the path planner is designed in a generic form which takes the robot’s specification *S* as an input. The experiments are carried out on a computer equipped with an Intel (R) Core (TM) i5-8250U CPU @ 1.60GHz 1.80GHz processor and 8GB RAM.

The proposed approach is compared with sampling-based methods RRT and RRT* [10]. Compared with the basic RRT and RRT* [9], the extended ones [10] take random samples on centerlines instead of randomly sampling inside the vascular model. The parameter specification is given as follows: the maximum number of samples to take before timing out is 4048, the probability of checking for a connection to the goal is 0.1, and the number of nearby branches to rewire is 32.

### Statistical analysis

The statistically significant difference between the proposed method and others will be evaluated via Kruskal-Wallis test in this work. It is a nonparametric test that does not assume a normal distribution of populations. The null hypothesis is that there is no significant difference between solutions using different methods. If the significance level $\alpha =0.05$, the null hypothesis is accepted having $p>0.05$. If $p<0.05$, the null hypothesis is rejected, which demonstrates that there is a significant difference between the proposed method with others.

**Fig. 5:**
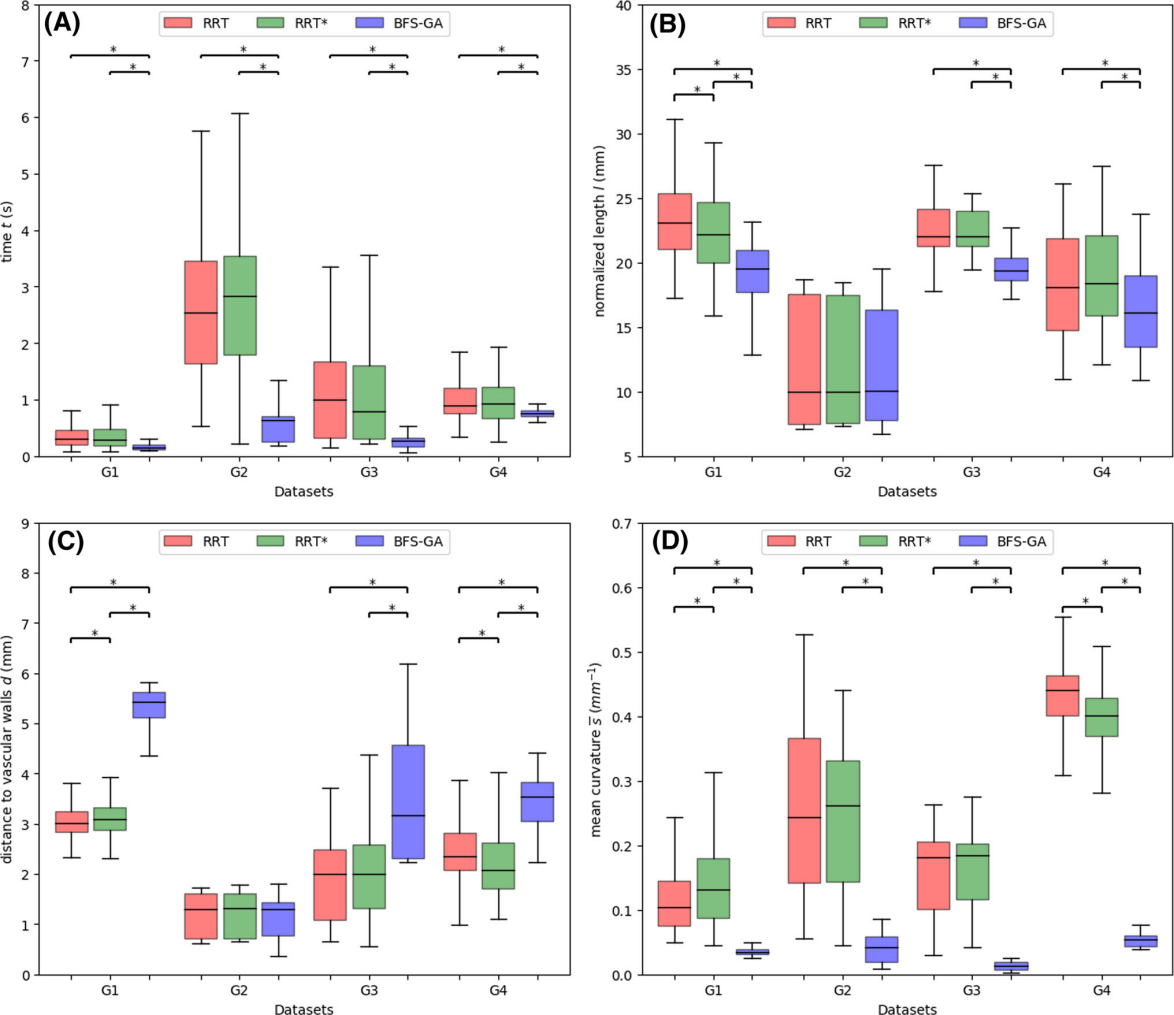
The performance comparison between the proposed method and sampling-based methods [10] according to (A) time cost, (B) path length, (C) distance to vascular walls, and (D) curvature. ($*, p<0.05$ using Kruskal-Wallis test)

## Results and discussion

The proposed approach is performed on our datasets and compared with sampling-based methods. Figure 5A shows that with respect to computational time cost, the proposed method has a smaller median and variance. In specific scenarios, where the blood vessels are slender and narrow, collision checking and avoidance of RRT series could take more time than continuous sampling along the vascular centerlines. More importantly, instead of considering the curvature constraint in the overall path planning which can be time consuming, we optimize curve segments in the local planner, for the reason that in most cases the curvature limitation would be respected except for some sharp turns along the centerlines. Therefore, the proposed method takes less computational time. Reducing computational time would help the path planner to be applied in path replanning. For example, the time cost on *G*1 is $191\pm 102$ms and the path replanning can achieve an updating frequency at $6.4\pm 2.3$Hz. Compared with serial threads processing in the proposed local planner, the speed of parallel threads processing improves noticeably. For instance, the time is reduced by 41% ($p<0.05$) when processing two threads in parallel on the dataset *G*4.

For path length, Fig. 5B shows that the proposed method has a smaller median value, while the variance is similar with the results of other methods. The random sampling property of RRT series leads to path points locating not always on vascular centerlines. Floating around the centerlines results in paths that can not be ensured to be the shortest one. To avoid this drawback, the proposed method adopts a BFS strategy within the vascular tree, ensuring the path solution to be the shortest one. Moreover, the local planner pushes the path points in the same direction away from the centerlines, avoiding bidirectional floating around the centerlines that increases path length.

Figure 5C shows that the proposed method increases the distance to vascular walls by keeping close to centerlines. It resulted not only from the sampling property analyzed in the previous paragraph, but also from the specified optimization criteria in the local planner. Figure 5D demonstrates that the curvature constraint is satisfied using the proposed method and the curvature median value is decreased. Specifically, the curvature constraint is respected in the local planner. The median value is also decreased by avoiding bidirectional floating around the centerline.

**Table 2:** The performance comparison regarding to success rate

*Method*	*G1*	*G2*	*G3*	*G4*
*RRT* [10]	0.980	0.760	0.890	0.910
RRT* [10]	0.988	0.740	0.890	0.950
*BFS-GA*	1	1	1	1

From Table 2, we can see that the success rate of our method is higher. As long as a feasible path exists, the proposed method is able to find it by navigating through the tree and optimize it locally. RRT series can not ensure a path could be found in a specific trail due to its incompleteness.

In short, the results show that the proposed method achieves a higher efficiency and better performance. It is further applicable for path planning in narrow passages for curvature constrained robots.

## Conclusion

In this manuscript, a fast two-phase path planning approach, named BFS-GA, is proposed for endovascular catheterization. Vascular centerlines were seen as a reference trajectory assisting catheterization in literature. State-of-the-art methods rarely consider the catheter curvature constraint. The presented approach is able to respect robot curvature constraints while keeping it close to the centerlines as much as possible. Moreover, researchers in literatures considered merely the constrained optimization problem along the overall path without reducing search space. In this work, we formulated and solved the optimization problem only for portions of the path and performed parallel optimization to shorten computational time. The limit is that it could lose accuracy in intra-operative interventions resulting from vasculature deformations and sensing uncertainties. Future works will concentrate on developing an accurate intra-operative path planner. A real-time path replanning algorithm based on a pre-operative path should also be proposed. Such an algorithm should consider additional factors like the unpredictable deformation of environments and the uncertainties of model sensing (e.g., the tip position and vascular model).

## Data Availability

Open source access of patient-specific images provided by SimVascular is available$^{1}$. https://simtk.org/projects/sv_tests
